# Hyper-Raman spectroscopy of non-proteinogenic amino acids

**DOI:** 10.1007/s44211-024-00698-1

**Published:** 2024-12-13

**Authors:** Tsung-Han Liu, Masanari Okuno

**Affiliations:** Department of Basic Science, Graduate School of Arts and Sciences, The University of Tokyo, Meguro, Tokyo 153-8902 Japan

**Keywords:** Hyper-Raman spectroscopy, Non-proteinogenic amino acid, Nonlinear Raman spectroscopy, Electronic resonance effect

## Abstract

We report 532-nm and 1064-nm excited hyper-Raman (HR) spectra of representative non-proteinogenic amino acids, including α-, β-, and γ-amino acids. Different from the common 20 proteinogenic amino acids, natural non-proteinogenic amino acids cannot be incorporated into proteins during translation, while they are indispensable as intermediates in many processes like biosynthesis and neurotransmitters. In 532-nm excited HR spectra, the COO^─^ symmetric stretching bands are commonly intense, and the NH_3_^+^ bands are clearly observable. In addition, based on the reported IR and Raman study, we found that some HR bands are IR-active but Raman-inactive. In contrast, HR signals with the 1064-nm excitation are much weaker than the 532-nm excitation. Nevertheless, we observed the COO^─^ scissoring band unexpectedly, much stronger than other bands with the 1064-nm excitation. Our results suggest that the electronic resonance effect plays a role in enabling us to detect HR signals in the UV region readily. We expect that this study provides a supplementary reference for HR spectroscopy of natural amino acids.

**Graphical Abstract:**

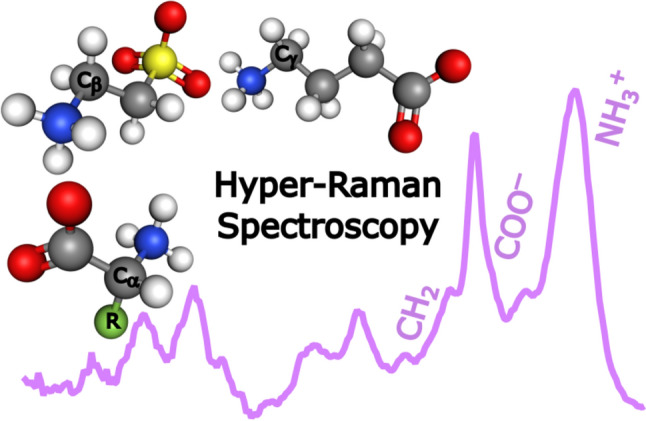

## Introduction

Vibrational spectroscopy is an essential tool to quantitatively and qualitatively analyze organic molecules. Based on vibrational spectra, molecular information on characteristic vibrational modes enables us to identify functional groups and skeletal structures and to investigate intermolecular interactions and sophisticated conformational changes under various physicochemical environments [1–3]. As the conventional approaches, IR absorption and Raman scattering spectroscopies have been widely applied to various molecules and composite systems. Because of their distinct principles and selection rules, IR and Raman spectra possess complementary active vibrational modes [1]. Hyper-Raman (HR) spectroscopy has recently received much attention as a complemental vibrational spectroscopy to IR and Raman spectroscopies. HR, Raman, and IR intensities originate from the molecular hyperpolarizability, polarizability, and dipole moment changes, respectively [4]. Both HR and IR signals derive from the odd orders of the electric dipole moment; the former is three, and the latter is one, while the Raman signal derives from the even order, two [4–6]. The distinct selection rules make HR spectra unique and different from IR and Raman spectra [4–6]. Therefore, HR spectroscopy can be an alternative analysis method to obtain the structural information on molecules [4].

HR spectroscopy has been very recently applied to various biomolecules, including *N*-methylacetamide (NMA) [7], amino acids [2, 3], polypeptides [8], proteins [2], nucleotides, and polynucleotides [9]. Previous HR work systematically studied Raman and HR spectra of 19 proteinogenic amino acids, except for phenylalanine, in aqueous solutions, suggesting that HR signals originating from the COO^─^ group are intense [3]. We have reported HR spectra of NMA, a model molecule for the peptide backbone [7]. In the fingerprint region, vibrational modes originating from the peptide backbone, including the amide I, II, and III bands, are clearly observed [7]. Following this previous work, we further demonstrated the application of HR spectroscopy to polypeptides [8]. The HR spectral information on the intensity order and the peak positions of the amide I, II, and III bands enable us to characterize their secondary structures [8]. These results strongly suggest that HR spectroscopy is suitable for studying the molecular structures of biomolecules.

Natural non-proteinogenic amino acids are not incorporated into proteins through translation but are indispensable in various aspects. They have been applied to peptide therapeutics due to their potential medicinal properties [10–12]. Some of them derive from proteinogenic amino acids during anabolism and catabolism as metabolites, or post-translational modification of the side chains of proteinogenic amino acids [12, 13]. These non-proteinogenic amino acids and proteinogenic amino acids are α-amino acids in a degree of similarity between their chemical structures. For example, in the urea cycle or the ornithine cycle, to convert highly toxic ammonia into urea, ornithine and citrulline occur as intermediates after the decomposition of arginine [14]. Ornithine also plays an important role in the biosynthesis of glutamate, arginine, and proline [15]. Citrulline is involved in de novo synthesis of arginine from glutamine and nitric oxide synthesis [14]. Hydroxyproline is produced by post-translational hydroxylation of proteins by the presence of a hydroxyl group attached to the C_γ_ atom [16]. As one of the major components of collagen, such as glycine and proline, hydroxyproline accounts for approximately 10% of all amino acids and plays an essential role in stabilizing the triple helical structures of the connective tissue collagen [15, 17–19]. Because hydroxyproline is specific to collagen, quantitative determination of hydroxyproline has been a widely used approach to evaluate the content of collagen in connective tissues [20, 21].

In addition to ordinary α-amino acids in which amino and carboxyl groups are bound to the same carbon atom, other amino acids like natural β- and γ-amino acid exist. As a sulfur-containing β-amino acid synthesized from methionine and cysteine, taurine (2-aminoethanesulfonic acid) is an amino sulfonic acid abundant in mammalian tissues [22, 23]. Because a sulfonic acid is a strong acid with a lower pK_a_ than a carboxylic acid, taurine is a zwitterion, H_3_N^+^(CH_2_)_2_SO_3_^─^ at physiological pH like the common amino acids [22]. As a representative γ-amino acid, GABA (γ-aminobutyric acid) is a common inhibitory neurotransmitter in vertebrate central nervous systems, opposite to glutamic acid as a major excitatory neurotransmitter and a precursor for the biosynthesis of GABA [24].

In this study, we are extending our interests to natural non-proteinogenic amino acids. We report HR spectra of representative ones, including α-, β-, and γ-amino acids, in aqueous solutions at neutral pH. Observed HR bands are assigned to vibrational modes by referring to the reported IR, Raman, and HR spectra of proteinogenic amino acids with similar chemical structures. We expect to provide information on vibrational modes of non-proteinogenic amino acids as a supplementary reference for further applications of HR spectroscopy to biomolecules essential in biological processes.

## Experimental section

### Samples

**Scheme 1:**
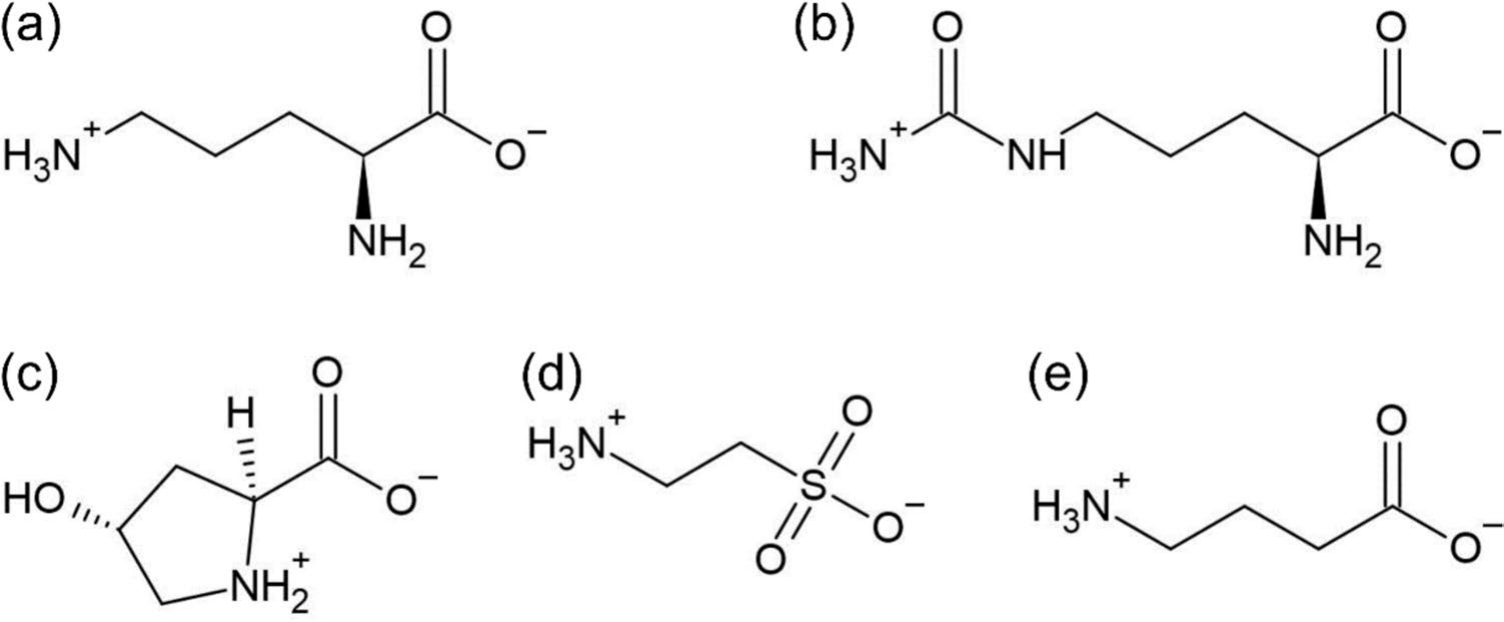
Chemical structures of l-ornithine (**a**), l-citrulline (**b**), l-hydroxyproline (4-hydroxyproline) (**c**), taurine (**d**), and GABA (**e**)

l-Ornithine monohydrochloride (≧98.0%, guaranteed reagent, Nacalai Tesque), l-citrulline (≧98.0%, guaranteed reagent, Nacalai Tesque), l-hydroxyproline (4-hydroxyproline) (≧99.0%, guaranteed reagent, Nacalai Tesque), taurine (≧98.5%, guaranteed reagent, Nacalai Tesque), and GABA (≧98.0%, guaranteed reagent, Nacalai Tesque) were used without further purification. To prepare their aqueous solutions at concentrations ranging from 0.5 to 1 M, tenfold concentrated phosphate buffered saline (PBS) at pH 7.2 (specially prepared reagent, Nacalai Tesque) diluted by ultrapure water (resistivity; 18.2 MΩ cm) from a water purification system (Purelab flex 3, ELGA) was used as a solvent to completely dissolve them at room temperature. Note that these amino acids are predominantly in the form of zwitterions in solutions around neutral pH (Scheme 1), because their pK_a_ values for the carboxyl and amino groups are far from the neutral pH [22, 25–30].

### Hyper-Raman spectroscopic system

Our HR spectroscopic system has been described in detail elsewhere [31–33]. The excitation light source was an Nd:YVO_4_ laser (Cepheus, Photon Energy; 1064 nm, 100 kHz, ∼15 ps). We mainly used the second harmonic light (532 nm) of the output of the laser source. For comparison, we also measured 1064-nm excited HR spectra. The average laser power was 150–180 mW for excitation at 532 nm, and ~ 1.1 W for excitation at 1064 nm. The vertically-polarized laser beam was focused by a lens onto a 1-cm square quartz cuvette inserted in a temperature-controlled holder (Luma 40, Quantum Northwest). All measurements were carried out at 25 ˚C. The HR scattered signals at 90˚ were collected by a lens and passed through an analyzer and a short-pass filter. The polarization directions of the incident laser light and the scattered light were set to be vertical. The scattered light was then introduced into a polychromator (MS3504i, SOL; 1800 grooves per mm, a 270-nm blaze grating for 532-nm excited measurements; 600 grooves per mm, a 500-nm blaze grating for 1064-nm excited measurements) and was detected by a CCD camera (iDus DU420A-OE, Andor). The slit width was set to 100 µm. The spectral resolution of our HR spectroscopic system was estimated to be about 10 cm^−1^. Exposure time was 600 s, and each HR spectrum is an average of 12 exposures. Wavenumber calibration was performed by using the emission lines of a handheld mercury lamp (GL-4, Panasonic) and a neon pilot lamp (BN-35, Sato Parts).

## Results and discussion

### HR spectra of non-proteinogenic α-amino acids (ornithine, citrulline, hydroxyproline)

**Fig. 1:**
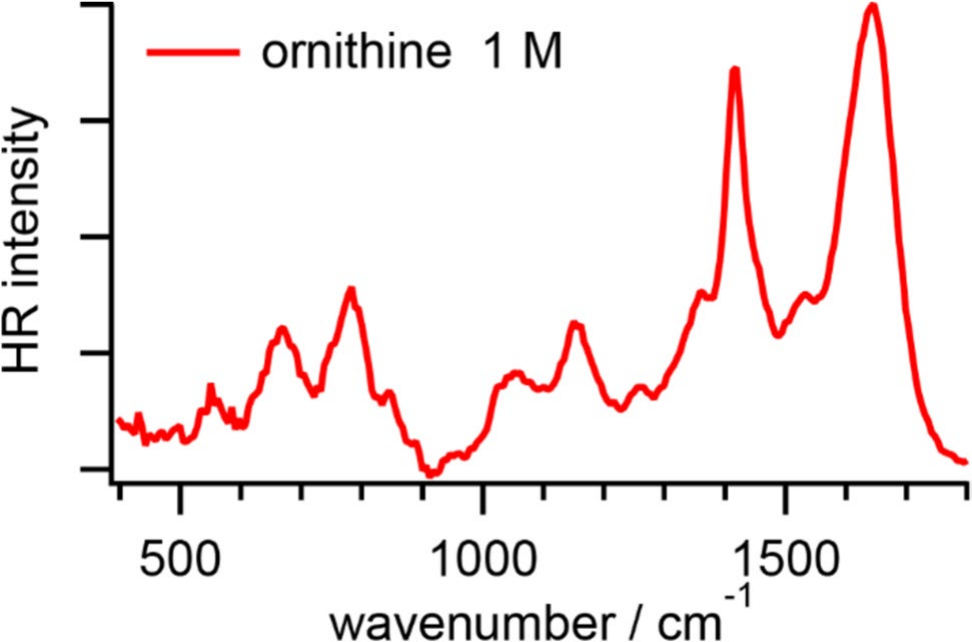
HR spectrum of l-ornithine in PBS at pH 7.2

Figure 1 shows an HR spectrum of ornithine in PBS. In the spectra of amino acids shown as follows, we have sequentially subtracted the baseline and the HR spectrum of PBS at pH 7.2 to minimize the HR signals originating from the librational and bending modes of water. We fitted the HR spectra with a combination of multiple Lorentzian functions for the intramolecular bands in the frequency range between 400 and 1800 cm^−1^. The fitted band positions are summarized in Table 1. In the HR spectra of ornithine in PBS, a broad band around 1640 cm^−1^ and a sharp band with a shoulder around 1420 cm^−1^ are relatively intense. Based on the reported Raman and IR spectra of solid-state l-ornithine nitrate and l-ornithine monohydrochloride, the former is contributed by the NH_3_^+^ asymmetric deformation and COO^─^ asymmetric stretching modes, and the latter is ascribed to the COO^─^ symmetric stretching mode [34–36]. Several tiny bands originating from the CH_2_ deformation, wagging, and twisting modes ranging from around 1200 to 1500 cm^−1^ are observable [34–36]. In addition, in the low-frequency region, some weak bands due to the COO^─^ wagging, deformation, and scissoring modes are found [34–36].

**Table 1:** Vibrational modes and frequencies (unit: cm^−1^) of ornithine

HR (in PBS)	IR (l-ornithine nitrate, solid) [34]	Raman (l-ornithine nitrate, solid) [34]	Raman, IR (l-ornithine monohydrochloride, solid) [35, 36]	Assignments
1641	1630	1637, 1605	1635, 1613	NH_3_^+^ as. def., COO^─^ as. str.
	1584	1540		NH_3_^+^ s. def.
1523, 1451	1505, 1484, 1448	1526, 1457	1490, 1465, 1448, 1420	CH_2_ in-plane def., CH def.
1416	1414	1414		COO^─^ s. str.
1354	1366	1349		CH_2_ wag.
1261	1286, 1244	1294, 1246, 1203	1292	CH_2_ twist.
			1249	CH_3_ twist.
1156	1147, 1126	1149, 1128	1201, 1150, 1104	NH_3_^+^ rock.
	1098, 1019	1097, 1021		CN str.
1048	1040	1051	1061, 1020	CCN as. str.
946	985, 927, 843	994	988, 938	CC skeletal str.
887	882	889	890	CCN s. str.
850	827	831		COO^─^ wag.
782	781, 754	780, 752		OCO def., CCO def.
	723, 710	724, 711		CH_2_ rock.
667	666	663	674	COO^─^ sci.
554	555	557		NH_3_^+^ torsion
	471, 445	463	459, 455	COO^─^ rock.

as. asymmetric, def. deformation, str. stretching, * s.* symmetric, wag. wagging, twist. twisting, rock. rocking, sci. scissoring, bend. bending

Around neutral pH, the chemical structure of ornithine, H_3_^+^N(CH_2_)_3_CH(NH_2_)COO^─^, is similar to that of lysine, H_3_^+^N(CH_2_)_4_CH(NH_2_)COO^─^ [3]. Consistent with the reported HR spectrum of lysine in PBS, the broad band above 1600 cm^−1^ is assigned to the NH_3_^+^ asymmetric deformation and COO^─^ asymmetric stretching modes. The sharp band at 1412 cm^−1^ is ascribed to the COO^─^ symmetric stretching mode coupled with the CH rocking modes [3]. Compared to the reported HR spectrum of lysine, our HR spectrum of the ornithine aqueous solution gives more fine structures assigned to vibrational modes of the CH_2_, COO^─^, and NH_3_^+^ groups below 1300 cm^−1^.

**Fig. 2:**
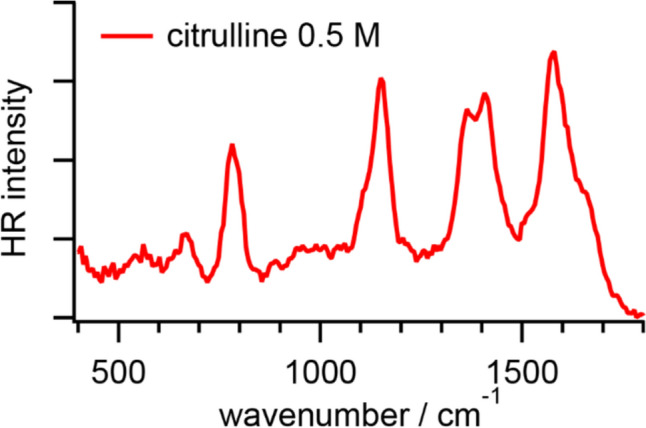
HR spectrum of l-citrulline in PBS at pH 7.2

Figure 2 shows an HR spectrum of citrulline in PBS. The fitted band positions are summarized in Table 2. Around neutral pH, citrulline is in the zwitterion form, H_3_^+^NC(O)NH(CH_2_)_3_CH(NH_2_)COO^─^ [28]. Although there are two amino groups in citrulline, only the one at the terminal can effectively alter itself upon pH changes [28]. In the HR spectrum of citrulline in PBS, there are four separated intense signals; a broad band around 1600 cm^−1^ with a shoulder, a split band around 1400 cm^−1^, a sharp band around 1150 cm^−1^ with a shoulder, and a relatively weak band around 780 cm^−1^. The broad band around 1600 cm^−1^ with a shoulder is ascribed to the COO^─^ asymmetric stretching modes and NH_3_^+^ bending modes [37]. The split band around 1400 cm^−1^ can be decomposed into two components; one around 1410 cm^−1^ and the other one around 1361 cm^−1^. The former is contributed by the COO^─^ symmetric stretching mode, and the latter can be assigned to the O–CH in-plane deformation mode based on the reported IR and Raman spectra of solid-state l-citrulline and its metal complex [37, 38]. The sharp band around 1150 cm^−1^ with a shoulder originates from the NH_2_ wag mode. The relatively weak band around 780 cm^−1^ is associated with the CH_2_ rocking and COO^─^ scissoring modes according to the reported IR and Raman spectra of solid-state citrulline [37]. In the urea cycle, citrulline is made from ornithine and carbamoyl phosphate, in which the carbamoyl group is partly identical to urea [14]. We notice that the band around 780 cm^−1^ is characteristic in our previous HR spectra of urea aqueous solutions [39], which has been assigned to the CO wagging mode from the reported IR spectra [40].

**Table 2:** Vibrational modes and frequencies (unit: cm^−1^) of citrulline

HR (in PBS)	IR (l-citrulline oxalate monohydrate, solid) [37]	Raman (l-citrulline oxalate monohydrate, solid) [37]	IR ([Cu(Cit)_2_]_n_, solid) [38]	Raman ([Cu(Cit)_2_]_n_, solid) [38]	Assignments
1629	1641		1636		NH_3_^+^ as. bend. [37], COO^─^ as. str. [38]
1576	1581, 1528, 1483	1486	1575, 1549		NH_3_^+^ as./s. bend. [37], NH_2_ sci. [38]
1410	1411	1431	1385		COO^─^ s. str.
1361	1346				O–CH in-plane def.
	1325, 1300	1315		1327, 1312	CH_2_ wag, CH_2_ vibration [37], CH_2_ sci. [38]
	1250			1279	C–O str. [37], COO^─^ s. str. [38]
	1197, 1103, 941	1200, 1100, 943			NH_3_^+^ rock.
1153	1143	1149			NH_2_ wag.
				1127, 990	CN str.
	982	994			CH_2_ rock.
	868	873			CC str.
	830	831			NH_2_ rock.
780	776	771			CH_2_ rock., COO^─^ sci.
	720	729			NH wag.
668	690	678, 615		682	NH_2_ out-of-plane bend. [37], COO^─^ bend. [38]
				567	CO rock.
	531				NH_3_^+^ torsion

For abbreviations, see Table 1

Citrulline has a similar structure to arginine, H_3_^+^NC(NH)NH(CH_2_)_3_CH(NH_2_)COO^─^. Indeed, the HR spectrum of citrulline in PBS is similar to that of arginine in PBS [3]. In the reported HR spectrum of arginine, a broad band with a shoulder appears around 1650 cm^−1^ is associated with vibrational modes of CN, CNN, and NH bonds; a split weak band around 1400 cm^−1^ is contributed by the COO^─^ symmetric stretching, CN out-of-plane scissoring, and CH bending modes; an intense band around 1180 cm^−1^ is ascribed to the CN stretching, NH rocking, NH_3_^+^ rocking, and C_α_C_β_ stretching modes; a tiny band around 725 cm^−1^ is assigned to the CN out-of-plane scissoring mode [3]. Distinct from the HR bands of arginine, we found that the band around 780 cm^−1^ is unique to citrulline as we mentioned.

**Fig. 3:**
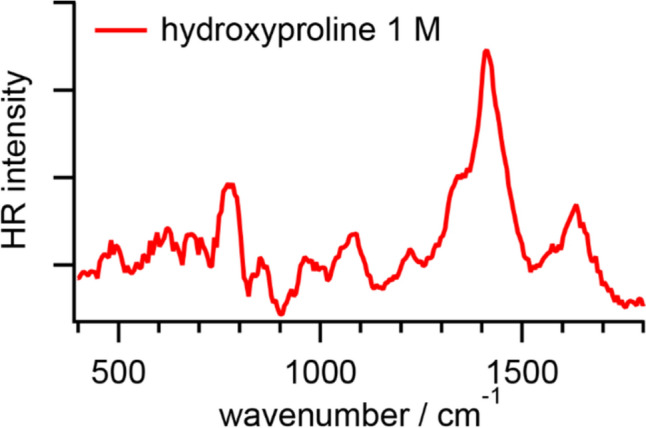
HR spectrum of l-hydroxyproline in PBS at pH 7.2

Figure 3 shows an HR spectrum of hydroxyproline in PBS. The fitted band positions are summarized in Table 3. An intense band with a shoulder and several fine bands are seen in the HR spectrum. According to the reported IR, Raman, and HR spectra of l-proline, the intense signal around 1410 cm^−1^ is ascribed to the COO^─^ symmetric stretching mode [3, 41]. A relatively weak band around 1630 cm^−1^ is assigned to the COO^─^ asymmetric stretching mode [3, 41], while the NH_3_^+^ scissoring mode and the water HOH bending mode may contribute to it at higher vibrational frequencies [4, 42]. In addition to weak bands originating from the CH_2_ bending vibration, some bands associated with the CC/CCN stretching modes and skeletal deformation are observed [3, 41]. Generally, HR spectra of hydroxyproline and proline in PBS are similar due to the structural similarity. In the low-frequency region, there are some tiny HR signals observable. Interestingly, the band around 612 cm^−1^ cannot be appropriately assigned based on the reported IR, Raman, and HR spectra of proline. In contrast, the vibrational frequency of the COO^─^ asymmetric stretching band in the HR spectrum of hydroxyproline in PBS may be slightly higher than that in the reported HR spectrum of proline in PBS.

**Table 3:** Vibrational modes and frequencies (unit: cm^−1^) of hydroxyproline

HR (in PBS)	IR (l-proline, solid) [41]	Raman (l-proline, solid) [41]	Raman (l-proline in an aqueous solution at pH 6) [41]	HR (l-proline in PBS) [3]	Assignments
1633	1619	1624	1616	1616	COO^─^ as. str.
(1448?)	1447	1451, 1441	1457	1451	CH_2_ sci.
1411	1402	1409	1411	1408	COO^─^ s. str., CO str.
1340	1340		1333	1330	CH_2_ twist.
1220	1225	1239			CH_2_ wag.
	1168	1173, 1164	1171	1178	CH_2_ twist.
1074	1083	1082	1090	1089	CH_2_ rock.
	1051	1056	1045	1040	CH_2_ wag.
971	979	994	992	987	CCN str., CC str.
857	860, 846	866, 842	864, 845	855	CH_2_ rock.
775	791	795	785	782	skeletal def.
686	688	698	681		COO^─^ sci.
612					
472	450	448	454	460	COO^─^ rock.

For abbreviations, see Table 1

### HR spectra of non-proteinogenic β- and γ-amino acids (taurine and GABA)

**Fig. 4:**
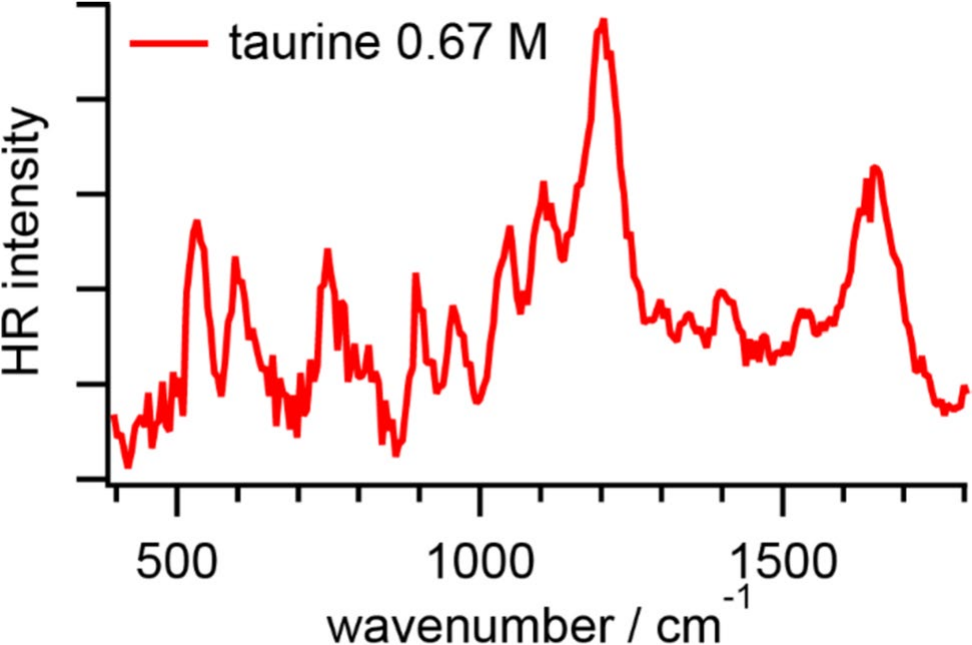
HR spectrum of taurine in PBS at pH 7.2

Figure 4 shows an HR spectrum of taurine in PBS. The fitted band positions are summarized in Table 4. The HR signal was relatively weak compared to other amino acids in this study. In the HR spectrum of taurine, a relatively intense band around 1200 cm^−1^ and several sharp but weak bands are observed. As a sulfur-containing amino acid, taurine has a sulfonic group instead of a carboxyl group. From the reported IR and Raman spectra of taurine in solid-state or aqueous solutions, the intense band around 1200 cm^−1^ is ascribed to the SO_3_^─^ asymmetric stretching mode accompanying the CH_2_ twisting mode, and a relatively weak band nearby around 1040 cm^−1^ is assigned to the SO_3_^─^ symmetric stretching mode [43–45]. The bands around 1650 and 1530 cm^−1^ are ascribed to the NH_3_^+^ asymmetric and symmetric deformation modes, respectively [43, 44]. The former band assigned to the NH_3_^+^ asymmetric deformation mode is relatively broad and its vibrational frequency observed in our HR spectrum is higher than in the reported IR and Raman spectra [43, 44]. Note that the contribution of water may still be involved in our HR spectra of amino acids to some extent because it is practically difficult to simultaneously minimize the HR signals originating from the librational and bending modes of water by simply subtracting the HR spectrum of PBS. Therefore, the HOH bending mode of water around 1650 cm^−1^ may contribute to the former band [4]. In addition, other weak signals related to vibrational modes of the CH_2_ groups, and the CC/CS bonds are observed [43, 44].

**Table 4:** Vibrational modes and frequencies (unit: cm^−1^) of taurine

HR (in PBS)	IR (taurine, solid) [43]	Raman (taurine, solution) [43]	Raman (taurine, solid) [44]	Assignments
1652	1615	1628	1613	NH_3_^+^ as. def.
1529	1512	1513	1588	NH_3_^+^ s. def.
1406	1458, 1427	1464, 1425, 1414	1459, 1427	CH_2_ sci.
1343	1344	1340	1344	CH_2_ wag.
1301	1304	1295		CH_2_ twist., NH_3_ rock.
	1250	1244	1259	CH_2_ twist., SO_3_^─^ as. def.
1203	1214	1207	1219	SO_3_^─^ as. str., CH_2_ twist.
	1179	1190	1181	SO_3_^─^ as. str.
1106	1112	1104	1110	NH_3_^+^ rock.
1043	1037	1046	1031	SO_3_^─^ s. str.
958	963	957	963	CC str., CH_2_ rock.
899	894	896	895	CH_2_ rock.
	848	840	846	CH_2_ rock., CN str.
		803		CS str. (*trans*)
755	743, 737	742	735	CS str. (*cis*), CH_2_ rock.
605	598	602, 588	593	SO_3_^─^ as. def.
532	534, 524	526	530	SO_3_^─^ s. def.
	467	458	471	NCC bend., SO_3_^─^ s. def.

For abbreviations, see Table 1

**Fig. 5:**
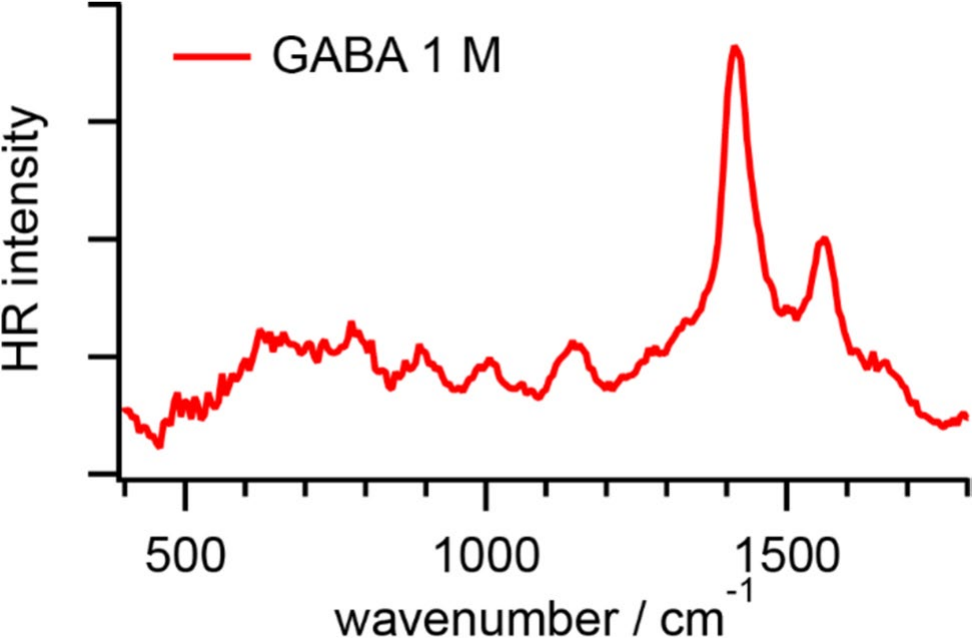
HR spectrum of GABA in PBS at pH 7.2

Figure 5 shows an HR spectrum of GABA in PBS. The fitted band positions are summarized in Table 5. In the HR spectrum of GABA, two intense and sharp bands appear around 1420 and 1560 cm^−1^, respectively. Based on the reported IR and Raman spectra of solid-state GABA and DFT calculations, the former is ascribed to the COO^─^ symmetric stretching mode accompanying the CH_2_ twisting, NH_3_^+^ twisting, CH_2_ deformation, CC stretching, and CH_2_ wagging modes, and the latter is assigned to the NH_3_^+^ wagging, COO^─^ asymmetric stretching, and CH_2_ scissoring modes [46, 47].

**Table 5:** Vibrational modes and frequencies (unit: cm^−1^) of GABA

HR (in PBS)	IR (solid) [46]	Raman (solid) [46, 47]	HR (l-alanine in PBS) [3]	Assignments
	1641			NH_2_ sci., NH_2_ twist.
1561	1562			NH_3_^+^ wag., COO^─^ as. str., CH_2_ sci.
		1470 [46], 1447 [47]		CH_2_ sci. [46]/bend. [47]
		1425 [46, 47]		CH_2_ sci. [46]/bend. [47]
1416		1402 [46], 1401 [47]	1417	CH_2_ twist., NH_3_^+^ twist., CH_2_ def., CC str., CH_2_ wag. [46], COO^─^ s. str. [47]
		1343, 1316 [46]		CH_2_ twist., NH_3_^+^ wag.
		1290 [46]		CH_2_ wag., CH_2_ bend., NH_3_^+^ wag.
1142	1169	1252, 1175, 1173 [46]		CH_2_ twist., CH_2_ wag., NH_3_^+^ wag.
	1124	1132, 1128 [46], 1126 [47]		CH_2_ twist., NH_3_^+^ wag. [46], NH_3_^+^ bend. [47]
1005	1007, 991	1011, 995 [46], 1009, 993 [47]	1007	CN str., CH_2_ bend./wag./rock., NH_3_^+^ twist., CC str. [46], CC str. [3, 47], CH_3_ rock. [3]
894	887	885 [46], 884 [47]		CH_2_ rock., CH_2_ twist., COO^─^ sci.
787	789	789 [46], 786 [47]	782	CH_2_ rock., COO^─^ sci. [46], CC str. [47], COO^─^ wag., CCC sci. [3]

For abbreviations, see Table 1

The chemical formula of GABA, H_3_^+^N(CH_2_)_3_COO^─^, is similar to those of l-alanine, CH_3_CH(NH_3_^+^)COO^─^, and β-alanine, H_3_^+^N(CH_2_)_2_COO^─^. The previously reported HR spectra of l-alanine in PBS suggest that the bands around 1420 and 1560 cm^−1^ mainly originate from the COO^−^ symmetric stretching and CC stretching modes, and the COO^−^ asymmetric stretching mode, respectively [3]. Interestingly, the band around 1560 cm^−1^ is not detected in the reported Raman spectra of solid-state l-alanine/β-alanine and l-alanine in solutions [48, 49], while it appears in the reported IR spectra of solid-state β-alanine [50]. The band around 1560 cm^−1^ has been consistently assigned to the COO^−^ asymmetric stretching mode [3, 50]. The results suggest that GABA is analogous to alanine, in between l-alanine and β-alanine.

### HR measurements under electronic nonresonant conditions

Ornithine, citrulline, hydroxyproline, taurine, and GABA are non-aromatic amino acids, meaning no absorption around 266 nm. In our previous HR study on model polypeptides, including poly-l-lysine, poly-l-glutamic acid, and poly-l-ornithine, HR spectra under the preresonant condition are similar to the reported UV preresonance and resonance Raman spectra [8]. In contrast, the previous HR study on proteinogenic amino acids using 532-nm excitation emphasized nonresonant conditions [3]. To study the excitation wavelength dependence of HR spectra from the samples in this study, we conducted HR measurements under excitation at 1064 nm.

**Fig. 6:**
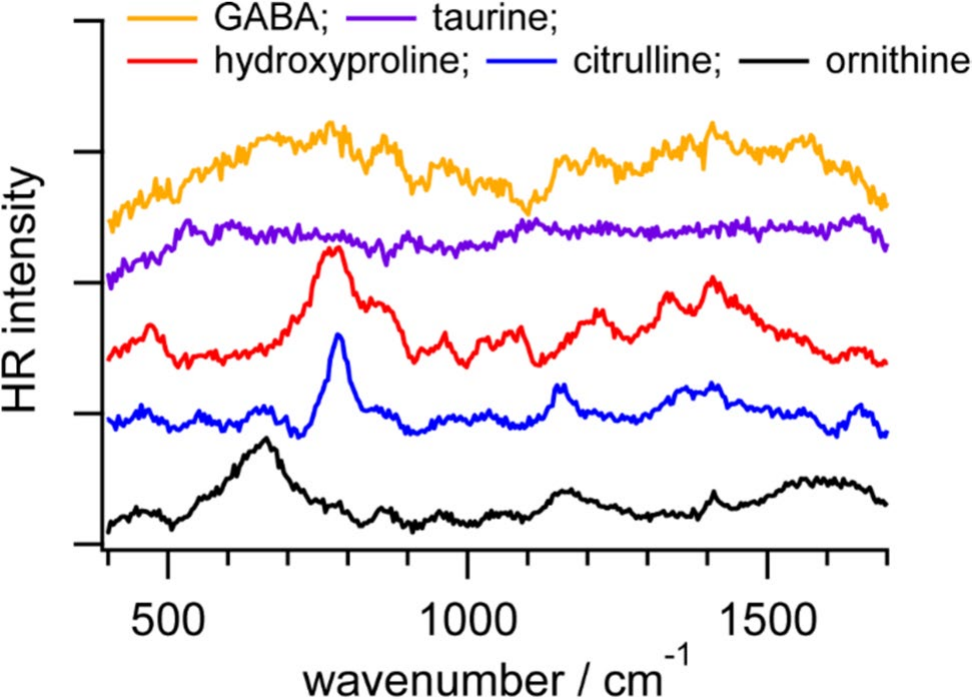
HR spectra of ornithine at 0.5 M (black), citrulline at 0.5 M (blue), hydroxyproline at 1 M (red), taurine at 0.67 M (purple), and GABA at 0.5 M (orange) in PBS at pH 7.2 measured under excitation at 1064 nm

Figure 6 shows 1064-nm excited HR spectra of ornithine, citrulline, hydroxyproline, taurine, and GABA in PBS. As mentioned previously, here we simply subtracted the baseline and the 1064-nm excited HR spectrum of PBS at pH 7.2, without normalization. Because the spontaneous HR cross-section depends on the excitation wavelength, 1064-nm excited HR signals should be much weaker than 532-nm excited HR signals [5]. Consistently, even if we increased the laser power to more than 1 W at 1064 nm, which is about five times higher than that at 532 nm, HR signals in the 1064-nm excited HR spectra are much weaker than those in the 532-nm excited HR spectra. If we measure an ornithine solution under the same laser power but at different wavelengths, the sharp band at 1412 cm^−1^ in the 532-nm excited HR spectrum of ornithine in PBS would be approximately one hundred times larger than that in the 1064-nm excited HR spectrum. Nonetheless, we found that the peak intensity ratios in the 1064-nm excited HR spectra differ from those in the 532-nm excited HR spectra. In comparison with the intense COO^─^ symmetric stretching band around 1410 cm^−1^ in the 532-nm excited HR spectrum of ornithine, the COO^─^ scissoring band around 670 cm^−1^ is unexpectedly observed in the 1064-nm excited HR spectrum of ornithine, which is also the case for citrulline. In the 532-nm and 1064-nm excited HR spectra of citrulline, in addition to the COO^─^ stretching and scissoring/bending bands, the characteristic band around 780 cm^−1^ is sharp and intense. In the HR spectrum of hydroxyproline, the bands related to skeletal deformation and other tiny bands ascribed to the CH_2_ wagging, twisting, and rocking modes are observed. However, the signal-to-noise ratios of the 1064-nm excited HR spectra of taurine and GABA were too low to identify any vibrational band because of some background signals. The spectral differences between 532-nm and 1064-nm excited HR spectra indicate that the electronic resonance effect plays a partial role in enabling us to efficiently obtain 532-nm excited HR signals of non-aromatic amino acids even if their electronic absorptions are in the far UV region [31].

## Conclusion

We report the 532-nm excited HR spectra of representative non-proteinogenic amino acids, including ornithine, citrulline, hydroxyproline, taurine, and GABA, dissolved in PBS at neutral pH. Almost all vibrational bands were appropriately assigned based on the reported IR, Raman, and HR spectra of proteinogenic amino acids with similar chemical structures. As a remarkable and common feature in the reported HR spectra of proteinogenic amino acids, the COO^─^ symmetric stretching bands around 1410 cm^−1^ are intense in our HR spectra of non-proteinogenic amino acids. In addition, vibrational bands ascribed to the NH_3_^+^ modes are observed in our HR spectra. Citrulline and GABA show HR bands ascribed to IR-active but Raman-inactive modes. Our measurements with the 1064-nm excitation indicate that it is necessary to employ shorter wavelengths to obtain HR spectra with moderate signal-to-noise ratios to analyze non-proteinogenic amino acids.

## Data Availability

The raw spectra that support the findings of this study are available within the paper and its Supplementary Information file. If any raw data files are needed in another format, upon reasonable request they will be available from the corresponding author.

## Abbreviations

HR spectroscopy: Hyper-Raman spectroscopy
Cit: Citrulline
G ABA: γ-Aminobutyric acid
PBS: Phosphate buffered saline
